# “What I Want You to Know”: Adult Women's Stories of Cleft Lip and/or Palate

**DOI:** 10.1177/10556656251398119

**Published:** 2025-12-02

**Authors:** Danielle McWilliams, Alan Hebben-Wadey

**Affiliations:** 1 Salomons Institute for Applied Psychology, 2238 Canterbury Christ Church University, Kent, UK

**Keywords:** cleft lip and palate, mental health support, psychosocial adjustment, quality of life, social support

## Abstract

**Objective:**

Evidence suggests that cleft lip and/or palate can have significant psychosocial impacts on adults’ lives beyond the end of the standard treatment pathway. Although some studies indicate gender differences, no research has specifically explored the experiences of women with cleft. This study aimed to explore the stories told by women about their adult experiences of cleft, for discussion informed by intersectionality theory.

**Design:**

Participants were interviewed using a narrative, photo elicitation approach. Each participant brought photos they felt represented their story to an unstructured interview, which was recorded and transcribed verbatim. Data were subject to narrative analysis.

**Setting:**

Interviews took place on Microsoft Teams video call, with photos shared on-screen throughout, and lasted an average of 94 min.

**Participants:**

Fourteen women born with cleft aged 20 to 72 living in the United States or United Kingdom took part.

**Results:**

Six overarching discourses were identified and presented alongside 14 individual narrative synopses, depicting converging stories told by each participant throughout their interview. These were (1) enduring, (2) hiding, (3) striving, (4) healing, (5) reclaiming, and (6) reconciling.

**Conclusions:**

Viewing cleft through an intersectional lens offers important insights into the lived and living experiences of adults navigating treatment and the ongoing psychosocial impact of cleft. The findings suggest that expectations and pressures placed on women by society are compounded by, rather than exist in parallel with, the ongoing impact of cleft.

## Introduction

Treatment of cleft lip and/or palate (“cleft”) is primarily surgical, with procedures carried out during infancy commonly referred to in medical and public discourse as lip and/or palate *repairs*^1^ (italics added). Cleft can impact an individual's speech, hearing, and physical appearance and requires multidisciplinary team treatment. The past decade has seen increased focus and attention on the residual emotional needs of adults within the cleft community, who perhaps do not feel as “*repaired*” as the medicalized lens might suggest. Adults have reported low mood,^2^ dissatisfaction with appearance,^3^ treatment anxiety and difficulties accessing further treatment,^4^ symptoms of traumatic stress,^5^ social anxiety, and challenges in intimate relationships.^6,7^ Recommendations to support adults with cleft have included greater access to psychology support,^8^ routine and early screening for emotional distress in clinic settings,^2^ targeted support and resources for individuals experiencing bullying or discrimination,^6^ and awareness among teams of the burden of invasive and often appearance-altering treatment.^4^

Many studies of the psychosocial impact of cleft on adults are quantitative and conducted within preset parameters such as *life satisfaction* and *treatment outcomes*, providing helpful foundations for understanding the potential needs of this previously understudied population.^9–11^ One study highlighted that for a group of adults, talking positively about their difference in childhood, the presence of close friends, feeling confident in talking about cleft, and the ability to feel comfortable in public places all positively impacted on their psychological adjustment.^11^ These latter associations illustrate a link between cleft and shame and sense of social safety. Indeed, results of other studies indicate the presence of shameful feelings among adults with cleft, with participants reporting a need to develop strategies to minimize their cleft in social spaces such as by controlling the angles from which they are seen by others,^3^ emphasizing other aspects of their public personas (such as being funny or working on their physical fitness)^12^ or by avoiding engaging with others altogether.^7^ Gilbert's^13^ conceptualization of shame posits that shame is an internal state that can arise from external pressures to conform, leading to direct attacks by others and the self if one cannot meet the prescribed social standards.

Some groups who are typically afforded less power in society and are often under more pressure to conform, such as women, are more prone to shame and internalized negative ideas about themselves.^14^ Furthermore, when one's need to be seen as socially attractive cannot be met, such as in the case of an individual who has been taught by their social context that their appearance and/or speech is unacceptable, shame persists and can lead to the types of avoidance and self-doubt already described in the cleft literature.^13^

As more qualitative research is conducted, a complex and often dynamic picture of coping, adjusting, distress, and the impact of societal expectations on cleft-affected individuals emerges.^15–17^ This highlights that differences within the adult cleft population are important, and moving toward more holistic, intersectional considerations of adults navigating their contexts, including but not limited to their cleft, could yield important insights for clinicians supporting individuals with cleft.

### Intersectionality Theory and Cleft

Intersectionality is the idea that characteristics that afford an individual a level of dis/advantage in society do not exist in isolation and can be compounded in a way which “creates obstacles that often are not understood among conventional ways of thinking.”^18^ Applying this to disability, Wickenden^19^ highlighted the gendered experiences of disabled individuals, whereby disabled women must contend not only with the stigma and objectification that so often comes with living in a body that looks “different” or disabled but also with oppressive assumptions about women's roles in society. Some women feel they have been denied credibility as women, given their disability which is the more prevalent identity, due to societal views on what constitutes femininity and these intersecting identities not being compatible.^20,21^

The impact that cleft can have on one's speech and appearance, coupled with evidence for cleft-related appearance dissatisfaction and longer-term psychological impact on adults indicate that these insights from intersectionality theory and research could be useful in understanding differences within the cleft community. In a study exploring a variety of visible differences, women reported finding it difficult to overcome a negative self-concept because of societal expectations and norms surrounding “attractiveness” and “femininity”^12^; their appearance delegitimized their claim to a female identity. Although research exploring explicit gender differences in the psychosocial impact of cleft is scarce, women with cleft have reported more appearance-related social anxiety and avoidance than men with cleft.^16^

### Gender and Cleft in Existing Research

Some quantitative studies have reported lower life satisfaction and higher distress in women with cleft than in men.^9,22^ Similarly, in a qualitative study of adults’ experiences with cleft, themes around being attractive and of needing to conform to what someone of their gender “should” look like were more prevalent in female participants than male. Women in this study also made more comments about the burdensome nature of their cleft throughout their lives, whereas men spoke more of being “better off” or “lucky” to have a cleft than women.^17^ Together, these findings may partially explain the finding that women with cleft have been reported to request and undergo more aesthetic lip and nose revision surgeries than men,^23–25^ possibly demonstrating a stark and tangible consequence of these gendered narratives. To date, qualitative research has not specifically studied adult women's narratives of cleft, such that these inferences about the roles of shame and intersectional identities might be explored further.

### Aim of Current Research

Considering the evidence that there may be a gendered experience of cleft, this project invited women to share their lived and living experiences of being a woman with a cleft. In doing this, it aimed to lay foundations for understanding if and how intersecting gender and disability/appearance identities, along with narratives of shame, may be present among this population. This information is intended to inform care within and beyond cleft teams.

### Research Question

What stories do women born with a cleft lip and/or palate tell about their lives and experiences of cleft?

## Methods

### Design

A qualitative design was employed, utilizing both photo elicitation and narrative interview methods of data collection. Participants were asked to collate photographs which told their story of living as a woman with cleft and were subsequently asked to relay this story in a life-story interview.

The researcher chose to combine narrative interviews with photo-elicitation methods to create a more holistic inquiry into women's lived experience of cleft. Narrative interviews offered an open platform for participants to share what felt most pertinent to their lives,^26^ rather than restricting discussion to predetermined topics such as surgery,^27^ emotional adjustment,^8^ treatment satisfaction,^4^ or appearance concern,^16^ which might have felt limiting or irrelevant. In this way, the approach encompassed the breadth of lived and living experiences and sought to approach challenges without imposing frames such as resilience or coping, in line with trauma-informed principles. Photo-elicitation complemented this by encouraging emotional and detailed storytelling,^28^ supporting memory, fostering dialogue,^29^ and disrupting researcher-participant power dynamics. By collating photographs, the participant can prepare, ask questions of their lives and develop ideas about their narrative before the interview begins,^30,31^ thus making participants the “experts.” Importantly, the researchers acknowledged that many participants’ prior experiences of photography may be in medicalized contexts,^32,33^ or avoidance of photographs due to appearance concerns.^3^ Using a visual methodology without necessitating pictures of the participants themselves was considered a counterweight to these possible prior experiences.

### Researcher Reflexivity

The lead researcher and first author, a woman with a cleft (disclosed in participant materials), was motivated to conduct this study not only by professional interest and knowledge, but by lived experience and engagement with the cleft community. Together, these highlighted gaps in trauma-informed, holistic care which took characteristics such as gender into account. Insider status shaped recruitment, rapport, and data generation: disclosure and shared language sometimes facilitated access and richer accounts, and brief self-referential anecdotes were used to build trust and connection.^34^ The researcher attended to the importance of balancing her own position as a facilitatory tool with privileging the unique stories of each participant, ensuring her position was a strength, rather than a limiter, to the project.

### Epistemological Position

Photographs and stories provide access to lived experiences and individual truths, which are shaped by their beliefs and unique contexts. The researchers take a social constructionist view of knowledge: participants’ accounts are coconstructed through interaction between participant, their experiences, and the researcher, with meaning produced relationally and interpretively with respect to situational factors, rather than discovered as objective fact or absolute truth.^35^

### Expert by Experience Involvement

Health research that actively involves Expert by Experiences (EBEs) is generally acknowledged to be more relevant and sensitive to the needs of those who are most likely to be directly impacted by the work.^36^ Within cleft research, calls to advance the field include an emphasis on engaging with EBEs at all stages of research.^37^ The researcher worked with an EBE, a woman with a cleft living in the United States, to review materials, design the protocol, support recruitment and discussing this manuscript, conference presentations, and other dissemination efforts. This was facilitated using MS Teams calls approximately every 8 weeks.

### Ethics

Ethical approval was granted by the Canterbury Christ Church University ethics committee (approval #ETH2324-0081). Written consent including permission to use images was obtained from all participants. Pseudonyms are used throughout this report. A debrief sheet including well-being advice was sent via email to each participant immediately after their interview.

### Procedure

#### Recruitment

Participants were recruited via social media advertisements which were promoted by the Cleft Lip and Palate Association (CLAPA)^38^ and Face Equality International.^39^ An information sheet was provided via email to eligible participants detailing the methods, protocol, and framing of the project (see Table 1 for inclusion and exclusion criteria).

**Table 1.** Inclusion and Exclusion Criteria.

Inclusion criteria	Exclusion criteria
Identifies as a woman/female	Under the age of 18
Born with a cleft lip and/or palate	Medically unsafe to collate photographs and participate in a call of up to 2 h long
Is able to converse in English	Self-identifies the prospect of sharing potentially upsetting memories and experiences as psychologically unsafe

#### Photography Period

After the initial call, participants were given 1 to 3 weeks to collate photographs which they could use to tell their story during their interview. This was deemed appropriate in leaving enough time to consider and produce the images while maintaining interest and engagement. Participants were not limited on how many photographs they should send or on whether they used preexisting or new photographs. The researcher did not look at photographs in the folders prior to the interview to avoid forming preconceptions of the data and to ensure the same procedure was followed for all participants.

#### Interviews

Interviews took place on Microsoft Teams video call, lasting between 61 and 118 min, with an average length of 94 min. The interview schedule allowed for open storytelling, conversation, and discussion (eg, “tell me the story behind this photo”).^40^ Where participants indicated that they wanted further support to start or explain an element of their story, the researcher asked open questions about elements of what had already been said, thus not introducing any material that had not been provided by the participant. Interviews were recorded and transcribed verbatim.

#### Participants

Information was initially emailed to 37 women with a cleft who expressed an interest in participating, with reminder emails sent after 5 days if no response was received. Fourteen responded and opted to take part. Participant characteristics are outlined in Table 2.

**Table 2.** Participant Characteristics.

Pseudonym	Age at interview	Ethnicity	Employment	Highest level of education	Relationship status	Cleft lip type	Cleft palate type
Rebecca	20 years 9 months	White British	Student + part time employed	A-Level	Never married—in a relationship	Unilateral	Complete
Beth	22 years 3 months	White British	Student + part time employed	Masters	Single and never married	Unilateral	Complete
Sophia	23 years 5 months	White American	Student	Masters	Never married—in a relationship	Unilateral	Complete
Taniya	32 years 4 months	Black-African	Student + full time employed	Masters	Married	Bilateral	Complete
Anna	38 years 11 months	White (other)	Full time employed	Bachelors	Single and never married	Unilateral	Complete
Claire	44 years 11 months	White British	Full time employed	Masters	Married	Unilateral	Complete
Iris	53 years 4 months	White British	Unemployed volunteer	Masters	Married	n/a	Complete
Rose	54 years 11 months	White American	Freelance	Bachelors	Never married—in a relationship	Bilateral	n/a
Lindsay	55 years 2 months	White American	Full time employed	Masters	Married	Unilateral	Complete
Sassie	58 years 0 months	White British	Full time employed	Masters	In a civil partnership	Bilateral	Complete
Gemma	58 years 6 months	White British	Full time employed	A-Level	Single and never married	Bilateral	Complete
Morag	64 years 3 months	White British	Freelance	Bachelors	Married	Bilateral	Complete
Mary	70 years 6 months	White British	Retired	Masters	Married	n/a	Complete
Annmarie	72 years 10 months	White American	Retired	Masters	Married	Bilateral	Complete

### Data Analysis

This project employed narrative analysis^41,42^ with analytic attention paid to both personal and sociocultural levels of meaning. This approach places an emphasis on coconstruction of stories and their meaning, with an assumption that neutrality is neither possible nor the aim in storytelling and hearing.^42^

Analysis aimed to understand how participants made sense of their lived experience and how their voices converged and differed as a group. Thus, the method also allowed for the identification of shared narrative forms: broader discourses that emerged across multiple accounts.

Attention was paid to the meaning participants attached to their images and how they used them to punctuate their stories. The project prioritized experiential narratives, valuing content and tone over adherence to form.^43^ To this end, the definition of “narrative” was held loosely, constituting an account shared by a participant which held personal meaning to them in relation to their cleft. The “discourses” were the ways in which these individual narratives could be seen within and across each other; shared stories.

The analytic process (Table 3) involved iterative readings and movement between individual and collective stories.

**Table 3.** Step-by-Step Process of Data Analysis.^a^

Step 1	Transcripts were each read in full following completion and notes made during the interviews were reviewed.
Step 2	Transcripts were reread and key thematic concepts were noted alongside poignant quotations.
Step 3	Transcripts were reread and notes were taken on the participant and story's positioning, including: - What was being said about the participant within the story - What was being said about the me as a researcher within the story - What the participant was intending the consequences of their story might be (Colbert et al., 2013)
Step 4	Transcripts were reread and broader sociocultural/ideological narratives were identified within the personal stories told.
Step 5	A matrix was produced for each participant to collate steps 2-4 (3 examples in Appendix L) (Lewis, 2019).
Step 6	The matrices were used to produce synopses for each participant, conveying key messages, events and an overall tone of interview.
Step 7	Synopses were sent to each participant to review and suggest changes. Feedback from participants was collated (Appendix M) and changes were made. Participants were also given the option at this stage to choose a pseudonym.
Step 8	A process of interpretive narrative analysis was conducted across participant narratives. Shared narrative forms and tensions were identified across accounts, guided by recurring patterns and discursive themes.
Step 9	Findings were written up.

a Although this is presented as a linear process, earlier steps were continually revisited to consolidate understanding and add observations.

## Results

The results below are supplemented by direct quotations from participants (denoted with “”, with ‘…’ to indicate shortening, [–] to indicate context. A narrative synopsis for each participant is provided first in Table 4, with the intention of preserving each individual's story in context, such that it can be interpreted alongside the proceeding overarching discourses below.

**Table 4.** Narrative Synopses.

Participant (pseudonym)	Narrative synopsis
Rebecca	Rebecca is a 20-year-old white woman born with a unilateral cleft lip and palate. She lives in the United Kingdom and works in media. Rebecca's story is one of navigating early adulthood and leaving behind the cleft treatment journey, having recently been discharged from her cleft team. Although she feels fortunate to have had excellent care and continual support from her mother, leaving her feeling confident much of the time about her cleft, she also told stories of self-consciousness about particular aspects of her cleft, such as her sensitive mouth being a barrier to eating and questions about a potential fistula in her palate. However, she shares a reluctance to return to treatment so soon, particularly due to the emotional and practical impacts that might have. Rebecca volunteers for CLAPA and loves spreading awareness about cleft and facilitating residential weekends for young people. She told stories of fun and friendship but also of healing and catharsis watching other young people be able to share experiences and view her as a role model in the way that she viewed adults with cleft when she was a child. While some days are better than others and she often finds herself checking her appearance, including her nose, lips, scar and teeth, Rebecca is proud of her experiences and her cleft journey, and hopes that, together, the adult cleft community can continue to advocate for easier access and awareness of adult treatment and general visibility of cleft in the mainstream.
Beth	Beth is a 22-year-old white woman living in the United Kingdom and was born with a unilateral cleft lip and palate. Having only recently finished cleft treatment and been discharged from her cleft team, Beth feels “free” in many ways from the difficult and restrictive experiences of appointments and surgery. A challenging appointment, resulting in her not being offered the surgery she wanted, led to a journey of self-acceptance, which Beth is grateful for, but nonetheless feels that cleft professionals could be more adept at listening to and validating their patients’ needs and hopes. With the support of her family, beloved dog and other young adults with cleft that Beth found and developed close friendships with through CLAPA, Beth embraces opportunities for advocacy work within the cleft community; a community she has become proud to be a member of, as she has gotten older. Beth does have complicated feelings that come with leaving treatment, such as a “love/hate” relationship with hospitals; environments which caused much childhood distress but also became familiar and provided structure. Beth's telling of her cleft story was punctuated with messages of hope and gratitude for the support she has had and the insight that her treatment has given her to eventually accept and love herself. Although she is now embarking on a new medical journey of navigating treatment for her hearing impairment, which came about as an indirect impact of her cleft, Beth told me she enjoyed that her life and thinking is no longer dominated by her cleft, which makes the difficult periods feel worth it.
Sophia	Sophia is a 23-year-old white woman with a unilateral cleft lip and palate, living in the United States. She is studying a graduate degree in occupational therapy; a career she feels aligns with her values of seeing not what is wrong with a person, but how a person can be supported to live their best life despite challenging circumstances. Sophia's cleft very much impacts her life, despite being finished with cleft treatment. She spoke of having learned to accept her cleft and view herself as beautiful, with the support of her long-term partner, but also of being aware that she is not “conventionally beautiful” which affords her a number of disadvantages as a woman. A theme of the first half of her interview was “milestones,” whereby Sophia spoke of achievements that most adults would not consider important, such as drinking through a straw or being flirted with by a stranger. Sophia also told stories of continually needing to advocate for herself from adolescence, even challenging cultural norms of health conditions as taboo, in order to be seen and treated appropriately. Her experiences of cleft treatment have left her afraid of hospitals and medical situations, which she has found ways to cope with and process but often finds that professionals and family members do not understand. Sophia also named some achievements which were important to her story of cleft, such as teaching herself to play the flute as an adult, despite being told at age 6 that she would never be able to due to her cleft palate. Sophia ended her interview by calling for professionals to be open-minded to the idea that adults with cleft carry their childhoods with them, so opening up dialogue about people's stories is far more helpful than assuming they are “sorted” just because cleft treatment may finish in adolescence.
Taniya	Taniya is a 33-year-old Black woman living in the United States and was born with a bilateral cleft lip and palate. A continual reflection which we returned to throughout Taniya's interview was that she was recovering from cleft surgery, which she had chosen to undergo as an adult at the time of her interview. She felt that this operation had been “different” and she was surprised and unnerved that she was not adjusting to the change to her appearance as quickly as she has done before. This gave rise to a powerful metaphor of feeling like “Mr Potato Head” head with removable and interchangeable parts, but never really feeling like she recognized herself. Central to Taniya's narrative are her husband and 2 young children, who she explained she was “never meant to have” due to growing up feeling different and that having her own family was out of her reach. Dating was challenging and anxiety-provoking, especially given the importance of appearance in first impressions, but she now does everything she can to model acceptance, empathy and self-compassion to her 2 young sons. Taniya is a fierce advocate for improving the insurance system in the United States, and campaigns regularly along with others in the adult cleft community, motivated by her own negative experiences of surgery coverage being refused due to an ignorance about the importance of plastic surgery for physical and mental well-being, and discrimination against adults who opt for cleft surgery as it is seen as “not essential.” Taniya identifies herself as a proud, cleft-affected, black, plus-size woman and aims to be a role model for all those in the cleft community and beyond who feel marginalized or misunderstood.
Anna	Anna is a 38-year-old white woman, living in the United Kingdom, who was born with a unilateral cleft lip and palate and chromosome deletion. Her chromosome deletion diagnosis came as a young adult following which a long battle with healthcare professionals to be listened to. Feeling invalidated by her cleft team and others whom she needed to support her meant that she often felt alone when navigating this chromosome diagnosis. Anna spoke of navigating treatment and surgery as an adult and the implications it has had at work, especially given societal taboos around plastic surgery and its association with vanity. As a result, she has had to fight feelings of undeservedness of time to invest in her health and treatment and finds herself working harder to “make up for” periods where she needs to be away from work for medical treatment. Having an understanding employer, being into fitness and having a supportive friendship group have all been very positive factors in helping Anna cope. She also reflected on how shame may have been present within her family regarding her conditions and how, despite having a loving family, this sometimes plays on her mind. Anna is now actively involved with CLAPA and has enjoyed the opportunity to give back. This has also allowed Anna to connect with other cleft-affected adults, which has been empowering as it represents finding a community of people with shared experiences.
Claire	Claire is a 45-year-old white woman living in the United Kingdom. She was born with a unilateral cleft lip and palate and is a senior manager working in education. Claire's story began with the idea that cleft has not had as large an impact on her life as she has seen it have on others’ lives but nonetheless spoke of compensating continually for aspects of her cleft that she fears judgment on. Claire described carefully cultivating the parts of her image that she has control over, such as her clothes, her preparedness for almost any situation and her speech. However, aspects of her life in which she has less control, such as her hearing or her frequent visits for specialist dental treatment cause her the most distress. Claire's story was one of overcoming, of finding, maintaining and giving hope and compassion all while growing to accept and be grateful for her life as it is. Claire's support and love for, and from, her family was a continual narrative thread throughout her interview. She cited the unwavering support and positive attitude of her parents as protective from the otherwise damaging messages she received as a child about needing to be “fixed” and holds hope that professionals are now more careful with their language. Claire has recently become curious about the adult cleft community and her participation in this project was personally meaningful to her as it encouraged her to notice and start piecing together the impact that cleft continues to have on her life.
Iris	Iris is a 53-year-old white woman living in the United Kingdom. She was born with a cleft palate and Pierre-Robin syndrome in the 1970s and she reflects in her interview about how care has moved on since then. Most notably, she spent the first 2 years of her life lying face-down strapped into a contraption to keep her jaw and tongue in the right place. Later on in her interview, she discusses receiving a diagnosis of C-PTSD for which she has learned strategies to manage noises and feelings of being “trapped.” Her assistance dog allows her to feel confident when out and on public transport and will help her to get away if she feels threatened. Iris is a dancer, teacher, musician and tutor for young people facing obstacles to their learning and believes that her experiences of being underestimated and fighting to have her voice heard have afforded her the empathy and determination to support those who “traditional” methods and systems cannot. Despite making changes to others’ lives, Iris has tried repeatedly to access cleft team support, especially for the psychological impacts of her early treatment, but has been unable to have a referral accepted. Her platform for advocacy is within development and participation groups, where she continues to campaign for more accessible adult cleft care, but is frustrated at the length of time and red tape barriers that are involved in facilitating change, while adults’ voices remain unheard and unsupported. Iris described her adult life with a cleft as a time where pieces of the jigsaw puzzle that make up her life and personality are slowly coming together as she learns more about trauma, the choices she makes about how to express herself and what works for her in navigating the constant pressures and demands of society on individuals who look and sound “different.”
Rose	Rose is a 55-year-old white woman living in the United States. She has a bilateral cleft lip and has also, in recent years, been the victim of a traumatic attack which left her with further facial injuries and scarring. She reflects on how the recent attack has highlighted in many ways the difficulties she already lived with regarding her appearance as a result of her cleft. Rose is a successful journalist and described this not being something that came naturally to her, as usually she prefers not to be seen by people or draw attention to herself. This is demonstrated in her interview when she talks about avoiding mirrors and cameras wherever possible and always feeling like an outsider, so much so that perhaps it is not worth trying to fit in, especially compared to people who are “conventionally attractive.” However, much of her narrative alluded to wanting the voices of people with cleft to be heard, especially adults for whom support may not have been available earlier on. Rose speaks of merciless childhood bullying still haunting her now and how she has developed a “no-nonsense” approach to advocating for herself which others might even find hostile. Rose finds comfort in her horses and is intrigued by the growing adult cleft community. Although Rose's experiences may have taught her to be wary of others’ intentions when positive opportunities do come along, this interview was important to her in recognizing how far she has come, as she commented that she may not have been able to be as open even 5 years ago; a reminder that the journey absolutely does continue beyond the end of treatment.
Lindsay	Lindsay is a 55-year-old white woman living in the United States, who was born with a unilateral cleft lip and palate. Lindsay describes how superstitious beliefs about the causes of cleft where she was raised, religion, media depictions of scarring, societal views of plastic surgery, and attitudes toward women and difference have all shaped her journey navigating cleft care and learning to accept herself. Her narrative was one of gratitude for the treatment she received through charity support in her younger years and of pride of where she has gotten to in life, having had to work hard to grasp and forge opportunities. She also reflected on small acts of empowerment, such as choosing a bright red lipstick to “give people something to look at” when they stare. However, Lindsay describes experiencing painful reminders that she is different, such as in seeing a photo of herself or—before she met her husband—experiencing rejection when dating. Although Lindsay has not had much involvement with cleft care as an adult due to cost in the United States and traumatic childhood associations making hospital treatment stressful, she described sometimes wishing her treatment outcomes had been “better,” and wonders what could have been. Wearing a mask during the pandemic gave her some insight into this, as she finally felt “normal” and that she could completely conceal her cleft for the first time. One day, Lindsay would like to tell her story through writing, and reflected on how perfectionism and feeling a pull to control and preempt others’ reactions has held her back from starting this. Lindsay finds comfort in the idea that her experience of care paved the way for more advanced treatment options and that her cleft has afforded her empathy and a determination to strive to do well in life.
Sassie	Sassie is a 57-year-old white woman who was born with a bilateral cleft lip and palate and lives in the United Kingdom. Sassie began her story by narrating the shame associated with her cleft in the context of her natural teeth, particularly when comparing herself to others or considering ways that she has been spoken to in medical appointments. She expressed a belief that health professionals simply do not seem to understand the magnitude of pain and trauma around cleft dentistry. Sassie discussed feeling that parts of her life are secret and best left concealed, from her obturator through to her designer lipstick, due in part to the judgments she perceives will be imposed upon her by others if they were revealed. Despite great success in her working life, Sassie has experienced always needing to work harder than others to be seen and heard, and has been discriminated against in the workplace. She explained her view that evolutionary influences drive others’ reactions to her, with women not wanting to be her friend in case it lessens their chances of finding a partner, and men making fun of her for trying. Central to her overall story was the effort that Sassie continually invests into ensuring that her cleft poses as little problem to others as possible, as this in turn helps her to feel less of a spectacle. Nonetheless, Sassie has found comforts in places and people that allow her peace to be herself, including her animals and her partner, who she described bring worth and meaning to her life and her sense of herself in the world.
Gemma	Gemma is a 58-year-old white woman, born with a bilateral cleft lip and palate living in the United Kingdom. Gemma has spent much of her adult life not involved with cleft services, and recently went back to specialist cleft care in 2022 following a particularly difficult interaction with her general dentist, who did not seem to understand the high-stakes involved with dental treatment, considering her obturator. This interaction left Gemma spiralling—a process she described many people would interpret as “overreacting,” but points out just how reliant her appearance, speech, eating and drinking are on her obturator, thus she was relieved to be able to find a cleft specialist restorative dentist who could offer her the care she needed. Gemma is a keen crafter, describes herself as “tomboyish,” has a fine attention to detail and often finds herself working harder than her peers and colleagues to feel satisfied in her work—all aspects of her life that she attributes to her cleft in some way, considering her childhood experiences of treatment, the need for distraction, a self-consciousness about her appearance and the associated assumptions others’ may make about people who look “different.” Gemma ended her interview by reflecting on her journey toward finally being able to be proud and accepting of her cleft and her place within the adult cleft community—a journey which has taken decades and helped by finding the support of CLAPA and appropriate MDT cleft care in the past couple of years.
Morag	Morag is a 64-year-old white woman living in the United Kingdom, born with a bilateral cleft lip and palate. Her story felt largely divided into 2 eras—before and after meeting a compassionate general dentist who helped her not only to reaccess cleft care as an adult after 40 years, but to accept her cleft rather than constantly trying to hide or minimize it, often to her own detriment when it meant not accessing the specialist dental care that she needed. Morag told stories of learning to overcome lingering associations from childhood and embrace the strategies she has developed, such as always serving soup with garnishes to avoid feeling that she was back on a postsurgery diet, and maintaining her passion for commuting by bike, having started cycling to avoid school-bus bullies. Although Morag describes herself as a private person, she is speaking out more about the needs of adults with cleft, highlighting the gaps in dental provision, particularly with privatization and elective cosmetic treatment becoming commonplace. Having worked hard to overcome the shame associated with wearing a denture, Morag spoke of it as an essential piece of equipment which she could not be without but carries a huge emotional weight, and ought to be understood more holistically by professionals. Morag finished her interview by asserting that she can now be proud of her story and of her coming to accept and want to share it. She hopes to continue her advocacy work and support other adults to access the care and information that they deserve from informed and compassionate professionals.
Mary	Mary is a 70-year-old white woman who was born with a cleft palate and lives in the United Kingdom. Despite difficult and painful experiences with hospitals and dentists as a child, Mary found solace in her adult career working as a therapeutic radiographer, where the validation of wearing a uniform, holding credentials and having good relationships with patients increased her confidence. Mary reflected on her experiences more recently with healthcare professionals, both dentists and hearing specialists, and expressed disappointment that neither seemed to know about cleft and why her care might look different to someone who does not have a cleft palate. Mary's dentist was not sympathetic to her discomfort relating to childhood experiences and, in particular, Mary felt that they were not sensitive to how vulnerable she feels with many people looking in her mouth without asking. Mary lives with a fistula in her palate and spoke about the inconvenience of food getting stuck and needing to blow her nose regularly, which both can draw unwanted attention, invite questions and exclude her from social environments. Mary names her husband and family as her biggest supports, and although she experiences regular reminders that she is “different” as an adult with a cleft, she believes that age has made her more resilient, grateful and accepting of her life the way it is.
Annmarie	Annmarie is a 72-year-old white woman born with a bilateral cleft lip and palate living in the United States. Annmarie is a motivated and passionate public advocate for widening access to cleft care in the United States and her story was full of examples of where she hopes to inspire others to live fulfilled lives and accept themselves for who they are. Annmarie and her sister were both born with clefts, but after sadly losing her younger sister at a very young age and with attitudes toward difference at the time not as progressive as they can be today, Annmarie described her cleft as very rarely spoken about in her family as it was too painful. Annmarie described her 20s as a time where she perhaps felt ashamed and wanted to hide her face from others, but decades of healing, building connections and a successful and meaningful career in advanced practice nursing gave her the confidence to work toward healing and self-acceptance. Proving wrong the people who doubted her and growing her self-esteem to the point where she can look people in the eye and challenge their judgment have been important achievements throughout Annmarie's adult life. Annmarie enjoys spending time in nature, particularly with butterflies, with whom she identifies strongly due to their metamorphic abilities, and finds that activities such as yoga, meditation and tap dance help her to overcome difficult thoughts and memories, particularly around rejection from others both in childhood and adulthood. Annmarie is happily married and the support her husband shows to her cleft journey, her healing and continual advocacy for others has been very important and validating for her, especially as a woman who is sure that she has been “overlooked” by potential partners in the past due to her cleft. Being able to see past first impressions, accept difference in others and build deep, meaningful connections with people are all traits that Annmarie holds close to her heart and has learned the importance of as a result of her own experiences.

Six overarching discourses were identified in the stories the participants told of being a woman with cleft. These constitute stories of Enduring, Hiding, Striving, Healing, Reclaiming, and Reconciling and are presented with examples of individual narratives that scaffold them. These discourses are presented in sequence out of necessity, but all were present throughout almost every interview, and the ordering of this section does not depict a linear process.

### Enduring

**Figure 1.**
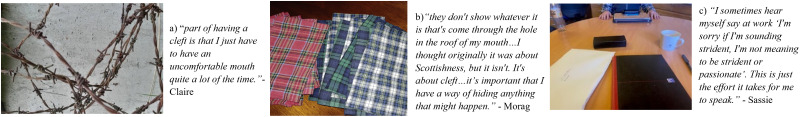
Participant quotations and accompanying images of (A) Barbed wire, (B) Tartan handkerchiefs, (C) work desk.

Twelve participants explicitly talked about the pervasive impact of childhood and ongoing medical experiences, sometimes decades later. For Claire, who describes herself as “*quite outgoing, I’m quite bolshy, I’m quite outspoken and…not afraid to be so,”* she struggles to make sense of her response to being in a dentist chair: “*I really regress…I go really like a little girl, like I cry and go all like meek. It's not me at all. And I hate it…I hate the person I am when I'm in the dentist chair.”* Mary reflects on the uncomfortable power imbalance between a dentist and their patient and how her dentist “*keeps saying ‘Your mouth's so small’ as if it's my fault…anatomically, we are a bit different. And we’ve been through horrible things in the past…she showed the student my mouth and didn’t even ask…you're made to feel like you’re some sort of freak. And most of the time they ask you to take your glasses off. And I know it sounds silly, but that makes me feel even more vulnerable.”* As the site of so much cleft treatment, dentists and dental work appeared as a source of discomfort in all but one (Taniya) of the participants’ stories (see Figure 1A). Anna describes that, even when treatment is less obviously invasive, being invalidated by professionals is exhausting and has had a lasting impact on the trust that individuals with cleft have to be able to place in their cleft teams “*I actually thought I had a chromosome deletion…the surgeon was just flicking through my notes and he laughed at me…literally no-one was interested…it definitely does make you lose trust in medical professionals. They make you doubt yourself too because like ‘surely, doctors know best.’”*

For Taniya, her recent surgery had highlighted the impact of her appearance changing so frequently throughout her life: “*you look in the mirror for so long and it looks a certain way…you get used to it. And then when it is repeatedly and drastically and in a span of 6 hours changed and you wake up and now like this is like your new normal…but it's not normal. It's not me. I don't hate it, but I am also like ‘I feel like Mr Potato Head with parts that don't belong to me.’ My nose doesn't feel like it's mine. It feels like I can whip it off and put another one on”* which she later added is a more difficult process to recover from each time.

Enduring others’ reactions to cleft seemed often more distressing than treatment itself. Narratives of being “*not conventionally attractive”* women were commonplace, and Sophia described missing out on “*milestones of femininity”* such as being flirted with or cat-called by men, which she recognizes as “*negative…but as someone who would never have that happen, it’d be novel. It's an aspect of womanhood which has never been accessible to me.”* For Sassie, her view of herself as unattractive is substantiated by other women's responses to her, which she explains is a direct result of dominant societal narratives about the roles women are expected to serve: “[other women are like] “*don't come near us, because if you're near us, then no men will want to approach us.” …because, you know, “we are fertile and beautiful and you are an anathema.””*

### Hiding

Concealing oneself in some way was a narrative across all participants’ stories. For some, this was hiding the physical features of cleft itself, such as Lindsay, Rose, Sophia, and Gemma, who cited the COVID-19 mask-wearing mandate as a way to hide their faces and to make them feel equal to those around them for the first time. While Morag and Rebecca both described feeling more confident in their appearance in adulthood, having a palatal fistula results in nasal regurgitation, which they find embarrassing and socially obstructive: “*after I've had some chocolate, I'll find myself getting a runny nose and its brown chocolate residue…it sounds absolutely disgusting and people go ‘eeeurgh.’”* Morag manages this by always carrying patterned handkerchiefs (see Figure 1B).

Employment presents unique challenges and triggers relating to navigating adulthood with a cleft for most participants. Following distressing childhood medical photography experiences as part of her cleft treatment and feeling ashamed of her appearance, Rose describes how she manages needing profile pictures for work opportunities: “*I just think for me black and white is not as unattractive. I think that a reader would just take a look at a black and white photo and not spend as much time studying it. I mean, I should have a professional headshot, but I want nothing to do with it.”* For Sophia, who is due to graduate soon and will be seeking employment, having a cleft means constantly minimizing her need for adjustments and concealing as much difference from her peers as possible: “*You're seen as a liability. They don't want to hire you because you're not going to be at work as often as a typical employee…you don’t talk about it, because you will end up disadvantaging yourself.”* This introduces the prevalent idea from many participants that cleft itself is not their primary concern, but the shame that they feel because of dominant narratives which disempowers individuals with cleft, is far more impactful. This is further explored by Anna regarding avoiding needing to explain to her work that she was taking time away for “plastic surgery”: “*When I was growing up, plastic surgery was a taboo…people don’t realize it's a medical specialty and plastic surgeons don’t just do people's boobs or whatever…in my old job, I had to put off surgery…I just didn't want to have that conversation with my managers.”*

Finally, for Annmarie, her story of hiding was one of feeling that her needs were being hidden as part of a wider social discriminatory practice of ageism. As an older adult with a cleft, Annmarie reflects on how invisible she feels that her cleft-related medical needs are, despite continually advocating for herself and others in her situation: “*it's like we're lost…I mean, it's really sad. I love what they're doing for children, but we need to look at all age groups.”*

### Striving

The pervasive feeling of needing to “measure up” to others, and perhaps to sociocultural expectations of women was described by most participants. Claire described carefully planning her outfits for each day because “*beauty bias is a real thing. People make assumptions about unattractive people, that they’re less likely to be intelligent…I think I give people a leg up by looking ‘the part’ and then they’re more likely to overlook my face.”* Similarly, some participants, such as Anna, described feeling a need to prove that they could have worthwhile qualities to employers, despite their cleft: “*with surgery…I need to work extra hard before and after to make up for it.”* For many, this was true in social spaces too. Sassie used an example of racial bias as a powerful parallel to how she feels entering a room and immediately feeling at a disadvantage, “*my stepson says that his blackness always enters the room before he does. And it's the same…my cleft is in the room, and if I'm really lucky…and I make people laugh, people start seeing me shortly afterwards.”* Sometimes, Sassie's striving to meet expectations of how women should be was even apologetic (see Figure 1C).

On a practical level, all participants described the effort that they have needed to continually invest in their cleft and/or dental treatment, far beyond the cessation of their original discharge from their cleft team. Fighting for adequate care of obturators was a source of particular stress for 6 participants, with Gemma stating “*if I couldn't wear it, I couldn't eat. I couldn't speak. I couldn't do anything…[dentists have] got no comprehension of how major that is”* and Sassie highlighting the trust that dentists demand regarding them “*The other thing about an obturator…what an absolute bloody ball-ache it is getting a new one…I don't like handing it over because people put it on the tissue on the side. I don't know where it is. It's so hard to get another.”* For Iris, the fight is simply trying to be seen by a cleft team: “*I don’t know what they do or what they don’t do but they’re rubbish at it so referrals got bounced every time…they don’t seem to know how…I need more help and I’ve tried so many times.”*

For most, a general sense of trying and fighting was present, across multiple themes. Taniya reflected on how cleft cannot and should not be held in isolation from other intersectionalities if people's stories of striving to overcome are to be fully appreciated: “*one of my college admissions questions was: tell us how you've overcome an adversity. And now I think like, “sure, but from what aspect? Being cleft affected? Being a woman? Being a black woman? Being a black woman who is also plus size?””*

### Healing

**Figure 2.**
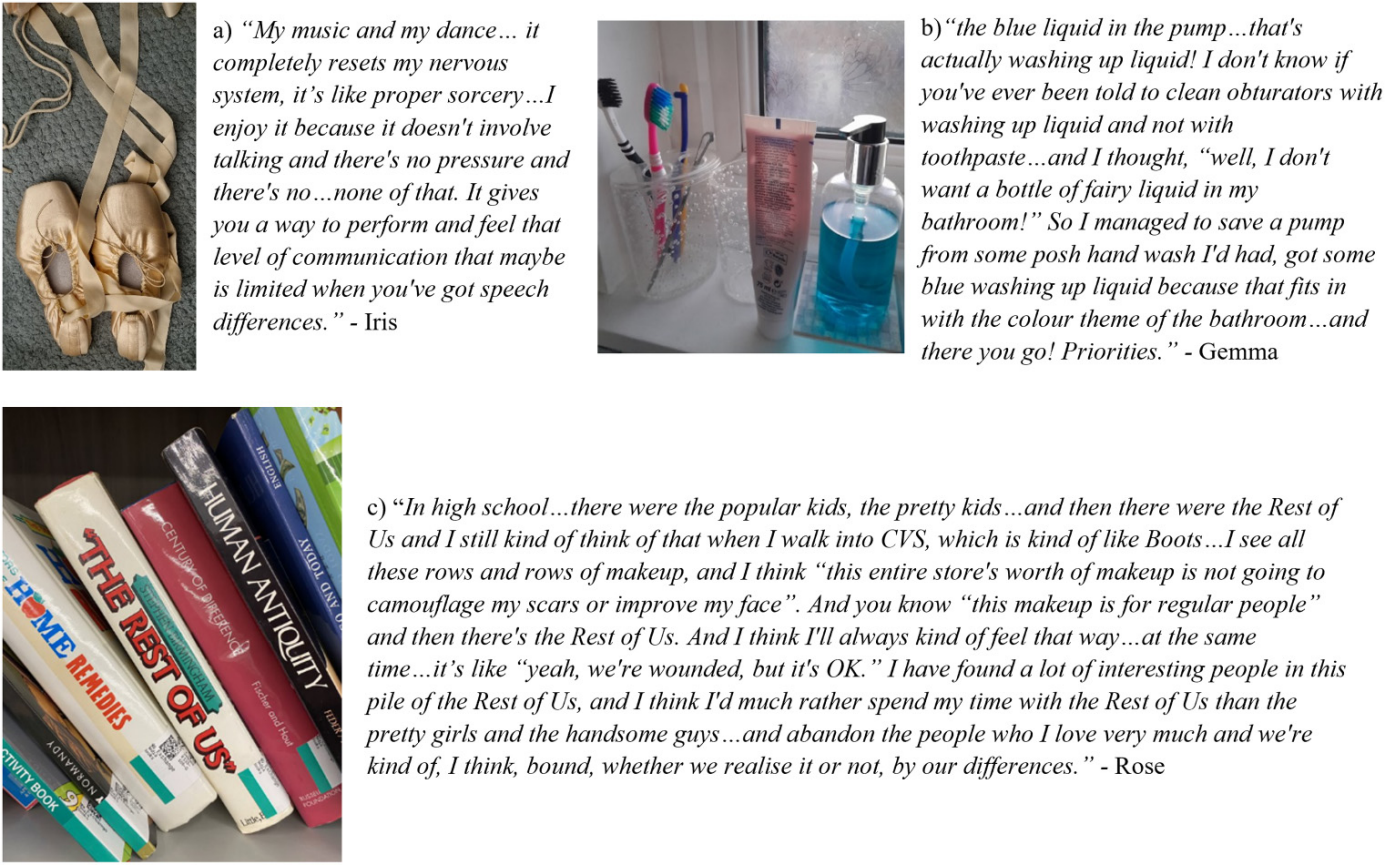
Participant quotations and accompanying images of (A) Ballet shoes, (B) bathroom shelf, (C) books.

By way of reprieve from emotionally- or labor-intensive aspects of coping with their cleft, all participants described aspects of their lives in which they sought refuge (see Figure 2A). Within these refuges of choice were stories of honoring and healing painful past experiences or beliefs relating to living with “difference.” Some of these were explicitly relating to treatment, such as Gemma always scheduling time to go to a café after appointments for cake in honor of a routine she had with her parents over 40 years prior, or Sophia's collection of photos of medical equipment or environments that were once a source of great stress, but she now looks at them to motivate herself as a reminder of how far she has come. For Rebecca, learning to ground herself and gain the perspective that “*‘my cleft doesn't have an impact on whether my train is going to be delayed…I'm just going to work like a normal person would…I do things the same way any other human being would’”* has been comforting.

For all participants, having close social bonds and feeling at peace with one's place among their social context were described as healing. Taniya reflects “*I am mummy to my children. I am my husband's wife. I don’t need anything else,”* having told stories of never believing she would be able to have a family of her own. However, some participants felt that the presence of loved ones conveyed a sense of normality to onlookers, such as for Sassie: “*Having the combination of dog and partner makes a massive difference when we go out and about. It's a normalizing thing, ‘Oh, she has a partner and a dog. She must be alright then.’”* This being seen as desirable as part of being “normal” manifested frequently when participants spoke about their relationships, specifically with intimate partners.

Finally, half of the participants told positive stories of meeting and befriending others with cleft, which for Beth, started reluctantly in adolescence where she remembers thinking “*why on earth would you want to sit in a room with everyone with the same problems as you…we don’t look good!”* but later embracing the community and reflecting “*If I was looking for support…my first port of call would be my friends that also have clefts…they understand and are open to listening and sharing on another level.”* However, while all participants mentioned encountering others with cleft, some, like Rose, felt less comforted about the idea “*I don't know if emotionally I can take a roomful of us!”* but later recognized that perhaps this was part of her journey toward healing, which could run parallel to Beth's story.

### Reclaiming

For most participants, finding ways to reclaim parts of their lives they feel less autonomous over, such as their medical care or others’ responses to their cleft, was important (see Figure 2B).

For Morag, who learned to ride a bike as a child to circumvent bullies on the school-bus, being a commuter cyclist is now a treasured part of her private and social identity which represents freedom, rather than escape. All but 2 participants discussed makeup as a route to reclaiming and somewhat sculpting what others would see of their lips and scars, but through different mechanisms. Lindsay's characteristic red lipstick holds the meaning that “*you know, if people are gonna look…I'm gonna give them something to look at,”* whereas for Sassie, her designer lipstick is “*just my private little thing…it's little private moment of ‘Here we go. It's OK to be out there.’”* For Iris, repeated surgery represented other people and processes claiming control over her face, compromising her feeling of connectedness to her own body. Piercings were a way of gaining “*control over what I looked like and who went in my headspace”* while acknowledging a wider sociocultural context that there is “*pressure on females about looking a certain way”* and that the approach of “*we’re not gonna look normal so lets not bother,”* allows Iris to claim ownership of her appearance beyond what others believe it should be. For others, reclaiming narratives were found in stories of proving wrong others’ limiting beliefs about their cleft. Annmarie, having been told she would never be a nurse due to her cleft, recalls realizing her worth by rejecting those who she felt rejected by: “*they equated…my appearance with a lower intelligence…once I got my [nursing] degree, I was asked to come back and teach…and I said ‘no thank you!’”* This reclaiming seemed to be of power and autonomy over one's ability, in a similar way to Sophia, who, in her 20s, taught herself to play the flute after being told as a child that she never could. Accepting and realizing that she must “*play flute differently than somebody else because of the shape of my top lip but I'm able to do it”* depicts a flexibility, as well as determinedness, in reclaiming previously denied experiences.

### Reconciling

For Rebecca, Beth, Taniya, Morag, Sophia, and Annmarie, publicly advocating for others with cleft represented a turning point in their lives, wherein a desire to hide was overridden by a calling to inspire, inform, and instil hope. Finding community and genuine allyship catalysed this shift, and for Beth there was a sense of choosing and being proud to declare her difference: “*I noticed a pride almost in my cleft, which I hadn't really felt before in that like, I'd always felt pride of being part of the CLAPA community but never…pride in my own cleft journey and kind of own it as, like, something actually really quite badass and cool.*” Morag, who only reports acknowledging her cleft in her 60s after meeting a compassionate dentist, says “*I can't be private if we’re going to achieve proper dental care and all the rest of it for other people like…us”* signifying being motivated by campaigning for change and finding community. In terms of treatment, Taniya concludes that there could always be more surgery, but her pursuit of more cosmetic work is now over, saying “*I have to ask myself who I’m doing this for. My children are not going to see me any different. My husband isn’t going to see me any different…I’m happy with where I am. How the rest of the world feels, its not my business. I don’t care.”* Among all participants, there was a sense of catharsis in the interviews whereby it was acknowledged that cleft remains “*in the room”* and its impacts will be sustained for as long as care remains a battle and dominant social narratives on what is it to be “attractive” and “normal” are prevalent. However, each individual had strategies of reconciling themselves with their difference in personally meaningful and emotive ways. Summarizing, Rose describes her journey to developing understanding and acceptance of her cleft and where she believes she “fits” in the world (Figure 2C).

## Discussion

This study aimed to explore the stories of adult women born with a cleft to better understand cleft from a gendered perspective. Within cleft, as a condition for which the primary treatment is medical and all treatment is managed within medical MDTs, little space has been made within research and practice for considering the intersectionality of living as an adult navigating cleft alongside living as a woman *within a society* holding established appearance and gender norms. Operations of shame can be inferred from broader literature suggesting that external pressures may become internalized, such that those who are or feel “othered” risk self- and other-attacks, reinforcing emotional distress and societal disadvantage.^13,14^

Qualitative research has identified that the factors impacting the psychological well-being of adults with cleft include social support,^7,11,44^ satisfaction with treatment outcomes,^11,44^ appearance-satisfaction,^7,11^ and tendency toward optimism.^11^ Furthermore, a comprehensive narrative review suggested the lifelong impact of cleft is “low,” though findings are complex and firm conclusions difficult.^45^ Within this study, the 6 discourses identified in participants’ stories depicted ongoing processes that appeared to be dynamic and constantly co-occurring rather than linear, which perhaps goes some way in explaining this complexity. Establishing a neat overall conclusion on “well-being” may be unreasonable when shame and enduring trauma coexist with healing and reconciling with cleft. These processes likely reflect systemic and sociocultural norms and power beyond individual control, and the ways that these are internalized and triggered in different ways. The current study builds on previous research findings that highlight that while distress is present in men and women with cleft, it is more prevalent and acute among women.^16,22^ It may indicate where dominant social narratives may be operating to maintain this distress, in addition to the ongoing adverse impacts of both relational and medical trauma. This was particularly prevalent within the “*hiding*” and “*striving*” narratives, where participants spoke of assumptions others make about their intelligence or desirability and their constant efforts to anticipate and overcome these.

Although all 6 narratives were identified across stories, developmental context may shape how they are emphasized. For example, Erikson^46^ posited that younger adulthood is characterized by identity and intimacy, midlife by caregiving or self-definition, and later life by reflection and legacy, evidence of which is seen within the stories in this project. Lifespan perspectives, such as Erikson's psychosocial stages, highlight how age-related roles and tasks influence meaning-making. From this view, variation across participants’ stories reflects the interplay between individual experience of cleft lip and palate and broader developmental and social contexts.

### (En-)Gendered Norms and Power

Centuries of literature have documented that being female generally affords less advantages than being male in Western societies and beyond. Women deemed attractive are more likely to be employed,^47^ succeed financially,^48^ and be successful in dating.^49–51^ “Beauty bias,” wherein physical unattractiveness, particularly in women, affords fewer opportunities, continues to be substantiated.^52–54^ Multiple studies of what constitutes female attractiveness conclude that visual symmetry and “softness”/femininity are most important and more determining of a woman's likeability than the equivalent in men, for whom nonphysical characteristics such as personality and perceived resources are privileged.^50,55^ Many participants reported being certain that their appearance altered others’ assumptions and behavior toward them and described themselves as in some way *less than* their noncleft-affected peers, particularly when speaking about social, intimate, or dating experiences. Indeed, both male and female laypeople rated images of faces with cleft as “less attractive” than those without, but men's responses were more extreme.^56^ The narratives “*hiding*” and “*striving*” depicted examples of where having a cleft can feel shameful, embarrassing, or isolating. Taking oneself away from others to adjust an obturator or manage nasal regurgitation were examples of this, where an awareness of cleft was egregious and obstructive.

Feminist and broader social justice movements throughout history have given rise to alternative narratives of reclaiming power, including around disability and facial difference.^57^ These assume a social model of disability, which rejects medical models of defects and abnormalities and adopts the stance that society, rather than biology, *dis*-ables individuals physically or mentally through discriminatory practices and norms.^58^ The narratives “*reclaiming*” and “*reconciling*” suggest a shift, or even rebellion against, oppressive societal messages that participants are “abnormal.” These narratives saw a marked shift away from shame and shaming self-talk. Most participants described an interest in advocating for others with cleft and in becoming involved in the broader cleft community, which may partly explain this omission of shame as individuals feel genuinely understood or equal to those with shared experience and understanding. Participants saw this as a positive part of their lives allowing them to find community, establish new norms, and embrace their cleft.

While most participants reported eventually coming to an acceptance of their cleft, the co-occurring emotional pain associated with being and appearing different was tangible throughout the interviews, as was the distinctly female voice of self-subjugation and bereavement of missed or inaccessible opportunities that not having a cleft may have afforded them.

### Recommendations for Clinicians

Considering not only the content, but the function of the stories told within this study could support clinicians in their understanding of their cleft-affected clients. Some existing research has concluded that with age comes self-acceptance and a reduction in care for others’ judgments among adults with cleft.^8,15,17^ The current study provides some support for this, but the coexistence of trauma, shame and concealment narratives within participants’ current, living experiences could indicate that more positive narratives serve as a protective defence against the emotional pain of the others. According to psychodynamic frameworks, establishing truths to justify adversity is a common mechanism by which people learn to live with uncomfortable or painful realities of their past or present beliefs and experiences.^59,60^ Therefore, remaining curious about the function of narratives to protect either the client or others could support cleft-affected clients’ stories to be heard and explored more thoroughly.

The presence of “*enduring*” narratives, particularly relating to dentists, medical care, and experiences of social stigma, indicate that adult cleft care must be trauma-informed, to meet the needs of those who need it. There is extremely limited research support and no guidelines for particular therapeutic models or interventions for individuals with cleft.^61^ However, these findings support the use of trauma-informed models that validate experience over diagnosis.^62^ Furthermore, they contribute to third-wave cognitive behavioral theories by evidencing the specific role of self-stigma in women with cleft, highlighting the potential for compassion-focused therapy (CFT) interventions to counteract entrenched shame in these individuals.^63,64^

The inherent power of being a medical professional should also be considered by clinicians in cleft teams. While they can and should be advocates for their patients in appointments and beyond,^65^ the environment may not feel approachable or safe for adults with cleft. This may hold truer still for women, for whom medical interactions are more broadly indicated to feature oppressive power relations requiring more active resistance than for men.^66,67^ Narratives of “*enduring*” and “*striving*” within participants stories provided examples of this power in action. Within therapy, his barrier could be acknowledged by explicitly naming what, or whom, the psychologist may represent for the adult client with cleft and taking steps to redistribute power in the therapeutic relationship, such as by meeting outside of the hospital, explicitly recognizing the expert knowledge of the client and/or taking longer to establish rapport before beginning change-orientated work.

Finally, despite recognizing significant emotional distress and, for some, a desire to seek mental health support regarding their cleft, most participants had never had contact with their cleft team regarding this. For some, this was because they were not aware of psychologists in cleft teams, others believed it was too late for them to seek help and at least one had a long history of rejected referrals. It is recommended that cleft teams find ways to reach out to adults, perhaps through colleagues in other cleft specialties and via dental and general practitioners in primary care services. Self-referral may also be a positive step in empowering adults to reaccess cleft care.

### Research Recommendations

Future research could build upon this study by mirroring the method and approach with men, exploring narratives of masculinity and power in relation to cleft. In addition, other intersectionalities, including but not limited to ethnicity, age, and sexuality, could be investigated in relation to adults born with cleft to promote and build momentum for more holistic understandings outside of the clinic room.

The narratives identified in this study could be used as a foundation for future clinical research. For example, “*hiding*” could be quantitatively explored further in terms of shame which could inform treatment decision-making and/or therapeutic interventions. Trials of therapies which seek to address shame, such as CFT, could contribute to the lacking evidence base for particular modalities in cleft care.

Finally, the interactions between participants’ stories and the researcher's own lived and living experience of womanhood and cleft was an important feature of the project. While it was not possible to evaluate this entirely within this report, data were collected about participants’ experiences of sharing their stories in this way, and it is hoped that it will inform future research. It would be particularly pertinent to consider the impact and experience of using photo elicitation methods with this group, given the salience of one's “image” in the context of visible difference and the role of photography in visible difference treatment. Future work may wish to explore the possibility of a therapeutic benefit of narrating lived/living experience using self-generated images.

### Strengths and Limitations

The open, narrative approach to interviewing and the multilayered data analysis process allowed for rich stories to be told and heard in this project. Given the relative lack of qualitative research conducted in this area, a more structured approach may have necessitated assumptions being made about where priorities for enquiry should lie, resulting in important nuances being missed. However, a limitation of this approach may have been that participants may have focussed more on explicit aspects of femininity had they been given more directive guidance.

A further strength of the project was the diverse age range of participants, which is likely due to the efforts employed to recruit through multiple channels. However, all but one participant was white, indicating that the project advertisement did not reach and/or feel applicable or interesting to those in the cleft community of the global majority. Additionally, many participants were highly educated and/or involved in advocacy, so were possibly more comfortable sharing stories than others less engaged in the community. Further research should aim to diversify participation using targeted recruitment if necessary to maximize the reach beyond those who are already engaged with organizations supporting and advocating for individuals with cleft.

With a shared language of the intricacies of cleft treatment and the ability to share, rather than solely hear experiences, it is likely that the researcher was able to ensure participants felt more comfortable telling elements of their stories than they would with someone who they did not feel could relate to them in this way. However, it was held in mind that participants may feel a need to withhold some aspects of their realities to protect the researcher's emotional well-being. There may have also been an assumption from participants that she already knew parts of their story and therefore did not need to be told, risking individual nuances being omitted. This was mitigated by assuming an open stance, acknowledging that there will be shared experiences which might include some impactful or emotive stories and discussion of thoughts and beliefs which may be different or similar to my own. The researcher reminded participants of their centeredness to their own interviews and was mindful of this when choosing what to share of her own story. With appropriate supervision, a robust reflexive diary, and utilizing prior knowledge and experience practicing self-care during this type of work, this influence served, rather than limited, the project.

Overall, this project employed a relatively novel method with a population primarily studied under a medical lens. It is hoped that by introducing intersectionality to cleft discourse in such an explicit way through the metaphorical and literal lenses of this group of participants will be meaningful in advancing the field further.

## Conclusion

Using a narrative analytic approach combined with photo elicitation, this study built on existing literature exploring the lived and living experiences of adults born with a cleft lip and/or palate, specifically focussing on women. Stories of enduring, hiding, striving, healing, reclaiming, and reconciling were shared and heard, converging in ways that defied the establishment of an orderly pattern or trajectory. Participants were clear that cleft remains a part of their life regardless of how much time has passed since the end of their prescribed treatment and emphasized the impact that others’ appraisals of them continue to have. Ongoing difficulties with accessing appropriate medical and dental care, friendships, dating and intimacy, employment, makeup, and living with the lasting impact of invasive medical procedures were all prevalent in the narratives. Being a woman who looks and/or sounds different in a society which often disempowers women and places expectations on their appearance and behavior was discussed as being exhausting by some, and unique ways of compensating and adjusting were explored. Clinicians and researchers should ensure they are aware of the intersections of cleft and gender in the context of adults’ ongoing experiences, such that their clients’ and participants’ realities can be fully heard in a trauma-informed and respectful way.
